# Exploring the Pathogenesis of Alzheimer Disease in Basal Forebrain Cholinergic Neurons: Converging Insights From Alternative Hypotheses

**DOI:** 10.3389/fnins.2019.00446

**Published:** 2019-05-07

**Authors:** Xu-Qiao Chen, William C. Mobley

**Affiliations:** Department of Neurosciences, University of California, San Diego, La Jolla, CA, United States

**Keywords:** Alzheimer disease, Down syndrome, BFCNs, Aβ, NGF, signaling endosome, Rab5

## Abstract

Alzheimer disease (AD) represents an oncoming epidemic that without an effective treatment promises to exact extraordinary financial and emotional burdens (Apostolova, 2016). Studies of pathogenesis are essential for defining critical molecular and cellular events and for discovering therapies to prevent or mitigate their effects. Through studies of neuropathology, genetic and cellular, and molecular biology recent decades have provided many important insights. Several hypotheses have been suggested. Documentation in the 1980s of selective loss of cholinergic neurons of the basal forebrain, followed by clinical improvement in those treated with inhibitors of acetylycholinesterase, supported the “cholinergic hypothesis of age-related cognitive dysfunction” (Bartus et al., 1982). A second hypothesis, prompted by the selective loss of cholinergic neurons and the discovery of central nervous system (CNS) neurotrophic factors, including nerve growth factor (NGF), prompted the “deficient neurotrophic hypothesis” (Chen et al., 2018). The most persuasive hypothesis, the amyloid cascade hypothesis first proposed more than 25 years ago (Selkoe and Hardy, 2016), is supported by a wealth of observations. Genetic studies were exceptionally important, pointing to increased dose of the gene for the amyloid precursor protein (APP) in Down syndrome (DS) and a familial AD (FAD) due to duplication of APP and to mutations in APP and in the genes for Presenilin 1 and 2 (*PSEN1, 2*), which encode the γ-secretase enzyme that processes APP (Dorszewska et al., 2016). The “tau hypothesis” noted the prominence of tau-related pathology and its correlation with dementia (Kametani and Hasegawa, 2018). Recent interest in induction of microglial activation in the AD brain, as well as other manifestations of inflammation, supports the “inflammatory hypothesis” (Mcgeer et al., 2016). We place these findings in the context of the selective, but by no means unique, involvement of BFCNs and their trophic dependence on NGF signaling and speculate as to how pathogenesis in these neurons is initiated, amplified and ultimately results in their dysfunction and death. In so doing we attempt to show how the different hypotheses for AD may interact and reinforce one another. Finally, we address current attempts to prevent and/or treat AD in light of advances in understanding pathogenetic mechanisms and suggest that studies in the DS population may provide unique insights into AD pathogenesis and treatment.

## Introduction

Alzheimer disease (AD) is the most common cause of dementia, accounting for up to 70% of cases (Apostolova, 2016). Clinical manifestations, which are insidious in onset, include memory loss and cognitive decline as well as behavioral dysfunction and failure to maintain function in activities of daily living (Scheltens et al., 2016). Patients progress from normal cognition to mild cognitive impairment (MCI) followed by increasing dementia severity (i.e., mild, moderate, and severe). While most cases are sporadic and occur late in life, other types of AD are recognized that present with earlier onset. Autosomal dominant forms of AD, which typically present in middle age, are due to mutations in the amyloid precursor protein (APP) (Bateman et al., 2011; Van Cauwenberghe et al., 2016), increased copy number for APP gene, and mutations in Presenilin 1 and 2, whose protein products regulate APP processing (Bateman et al., 2011; Van Cauwenberghe et al., 2016). APP, a type 1 transmembrane protein, is processed by sequential cleavage via either β- or α-secretase to produce the C-terminal fragments, β-CTF (or C99), or a-CTF (or C83), respectively. C99 is then cleaved by γ-secretase to yield the APP intracellular domain (AICD) and Aβ peptides of varying length; C83 cleavage yields the AICD and the P3 peptide (Thinakaran and Koo, 2008). A large body of evidence points to a pathogenic role for APP and its products in AD (Selkoe and Hardy, 2016). Down syndrome (DS), the most common genetic cause of AD, is due to trisomy for all or part of a third copy of chromosome 21. It is typically associated with the presence of a number of abnormal clinical phenotypes, including characteristic changes in craniofacial anatomy, and is universally marked by mild to moderate intellectual disability. The clinical presentation of dementia follows a course similar to AD, with problems with recall, explicit memory, and receptive language function before frank dementia. In those aged 30–40 the process may feature changes in behavior and personality (Ballard et al., 2016). Almost all adults with DS develop AD-like neuropathology by the age of 40. More than 50% display progressive cognitive impairment, leading to a diagnosis of dementia (Hithersay et al., 2017). The cause of death in DS is dementia in about 70% of cases (Hithersay et al., 2018). Given these findings this disorder is now termed AD in DS (AD-DS). The APP gene is present on chromosome 21 and is thus triplicated in DS (Megarbane et al., 2009; Ballard et al., 2016; Antonarakis, 2017). Correspondingly, the majority of adults with DS show increased expression of the gene with increases in the levels of the full length APP protein (Fl-APP) and all of its products (Prasher et al., 1998; Korbel et al., 2009; Wiseman et al., 2015; Ballard et al., 2016). Importantly, data are compelling that increased APP gene dose is necessary for AD-DS (Prasher et al., 1998; Korbel et al., 2009; Doran et al., 2017).

The neuropathology of AD manifests several cardinal features, including neuritic amyloid plaques and neurofibrillary tangles (NFTs) (Serrano-Pozo et al., 2011a). These markers are present in all types of AD, including AD-DS (Ballard et al., 2016). Amyloid plaques mark the extracellular accumulation and deposition of Aβ peptides, while aberrantly folded, and abnormally phosphorylated isoforms of the microtubule-associated protein tau serve as the principle constituent of NFTs. Both amyloid and tau pathologies show characteristic regional distributions and temporal patterns of evolution. Amyloid deposition begins in the frontal, temporal, and occipital lobes with later spread to hippocampus, the entorhinal, insular and cingulate cortices and amygdala; this is followed by involvement of several subcortical regions, including basal forebrain cholinergic nuclei, and later the substantia nigra, several brainstem nuclei, and the molecular layer of the cerebellum (Thal et al., 2002). While Aβ deposition is a prominent feature of AD pathology, studies do not support a role for plaque deposition in the temporal progression of dementia (Arriagada et al., 1992; Bierer et al., 1995; Giannakopoulos et al., 2003). Deposition of NFTs also follows a stereotypical spatiotemporal pattern, but one that is different than that for amyloid plaques. Braak and Braak defined six stages (i.e., Stages I-VI) in NFT involvement (Braak and Braak, 1991). The pattern features initial involvement of medial temporal cortex; later there is involvement of most of neocortex but with relative sparing of sensory, motor, and visual cortex. In contrast to plaque burden, the progressive involvement by NFTs is strongly linked to increasing cognitive deficits, with respect both the severity and duration of dementia (Arriagada et al., 1992; Bierer et al., 1995; Gomez-Isla et al., 1997; Giannakopoulos et al., 2003).

While for many years pathogenesis theories focused on amyloid plaques and tangles, an increasing body of evidence points to toxic Aβ and tau oligomers as having a primary causal role in the induction and spread of pathology and neuronal dysfunction and loss (Shafiei et al., 2017; Cline et al., 2018). In addition to plaques and tangles, other neuropathological hallmarks of AD include synapse loss and neuronal loss (Ball, 1977; Whitehouse et al., 1981; Dekosky and Scheff, 1990; Scheff et al., 1990, 2006, 2007; Scheff and Price, 1993; Gomez-Isla et al., 1996; Serrano-Pozo et al., 2011a; Andrade-Moraes et al., 2013; Calderon-Garciduenas and Duyckaerts, 2017) whose significance for disease manifestations is obvious. In addition, there is granulovacuolar degeneration (GVD), Hirano Bodies (Ball, 1977, 1978; Xu et al., 1992; Hirano, 1994), astrocytosis and microgliosis (Serrano-Pozo et al., 2011b). Importantly, changes in early endosomes are also manifestations in early stages of AD and AD-DS (Chen et al., 2018). Deficient endosomal transport of neurotrophic signals may play an important role in pathogenesis, as supported herein (Chen et al., 2018).

## Selective Vulnerability in Ad: Lessons From Parkinson’s Disease

Deciphering the pathogenesis and discovering treatments for AD has benefitted greatly by defining pathological manifestations in selectively vulnerable neurons. Studies in Parkinson’s disease (PD) provided a model for pursuit of selective vulnerability of AD. PD is a movement disorder whose cardinal features include bradykinesia (slowness of movement), rigidity, tremor at rest, and impairment of gait. The evolution of the dopaminergic hypothesis of PD, and its impact on therapeutic drug discovery, is a fascinating lesson in disease neurobiology (Fahn, 2018). PD was first described clinically by James Parkinson in 1817. More than 100 years would intercede before several key discoveries would guide the elaboration of the dopaminergic hypothesis and development of effective PD treatments: (1) the synthesis of dopamine (DA) in the 1910s; (2) in the late 1930s and 1940s, recognition of DA as an intermediate in the catecholamine synthetic pathway leading to norepinephrine and epinephrine (Blaschko, 1942); (3) documentation of its presence in brain and mapping its presence in several neuronal populations, including nigro-striatal DA neurons by Carlsson, Hokfelt, Fuxe, Dahlstrom, and colleagues in the late 1950s and 1960s (Carlsson et al., 1958; Anden et al., 1964, 1966; Dahlstroem and Fuxe, 1964; Fuxe, 1965a,b) (4) evidence for reductions in DA in the brains of patients with PD, including the substantia nigra, in the early 1960s (Ehringer and Hornykiewicz, 1960); (5) the demonstration in 1965 that neurons of the substantia nigra pars compacta were the source of DA in striatum (Poirier and Sourkes, 1965); (6) in the same year, evidence that lesioning the substantia nigra resulted in loss of DA in striatum (Poirier and Sourkes, 1965); and (7) increasing recognition during the same and later years that selective degeneration of DA-synthesizing neurons of the substantia nigra pars compacta is a fundamental neuropathological feature of PD (Fahn, 2018). These and related events motivated treatments to restore DA in PD patients. Cotzias and colleagues published the first definitive report of the utility of L-3,4-dihydroxyphenylalanine (L-Dopa, the precursor to DA) therapy, documenting its value in improving akinesia, rigidity and tremor, with sustained improvement ranging from moderate to dramatic in most of the 28 patients treated (Cotzias et al., 1969). While there is much yet to learn about the pathogenesis of PD, L-Dopa, and DA-based treatments continue to serve as the gold-standard of symptomatic treatment (Fahn, 2018). Thus, successful treatment was built on an approach that linked the loss of a specific population of neurons and their neurotransmitter with efforts to restore neurotransmitter levels.

## The Cholinergic Hypothesis: From Alzheimer’s Description to Treatment

Studies leading to the cholinergic hypothesis of age-related memory loss can be viewed in the context of those supporting the development of dopaminergic hypothesis. Key questions included: (1) is there a neurotransmitter whose actions critically support memory; (2) if so, are there changes in that neurotransmitter in disorders of age-related memory loss, most importantly in Alzheimer disease (AD); (3) if so, what neurons are responsible for producing the neurotransmitter; (4) do these neurons undergo degeneration in AD; and (5) will replacing the neurotransmitter confer symptomatic improvement. For AD, the first clinical description was that of Alois Alzheimer who described at a scientific conference in 1906 the case of a 51 year old woman, Auguste Deter, who presented in 1901 with reduced comprehension and memory, confusion, disorientation, changes in language including paraphasic errors, and perseveration, as well as psychiatric manifestations including paranoia and hallucinations (Maurer et al., 1997). She survived for 5 years. In 1907, in his full paper on the case Alzheimer noted that in addition to brain atrophy, silver staining methods showed “numerous small military foci,” now known to be amyloid β (Aβ)-containing plaques, and that within cells “there stands out one or several fibrils due to their characteristic thickness and peculiar impregnability,” the lesions we now refer to as neurofibrillary tangles. These findings were reconfirmed when the original slides were reexamined in the late 1990s (Maurer et al., 1997; Toodayan, 2016). The disorder was first named AD by Kraepelin in 1910 who indicated that the “anatomical findings suggest that we are dealing with a particularly serious form of senile dementia” but nevertheless noted that ‘this disease sometimes starts as early as the late forties” (Maurer et al., 1997). Subsequent years confirmed the existence of AD as a distinct entity and as the leading cause of dementia in the elderly (Reitz and Mayeux, 2014) with a worldwide prevalence of ∼4% of those greater than age 60 and an estimated number affected globally of 24 million (Calderon-Garciduenas and Duyckaerts, 2017).

As for PD, decades elapsed between the clinical description of AD, and the first effective treatment. But the focus on a possible role for acetylcholine in AD followed a somewhat different path than for DA. Indeed, already by the 1920s Otto Loewi had defined acetylcholine (ACh) as a neurotransmitter released by the vagus nerve to act on the heart to reduce its frequency of contractions, thus identifying ACh as a neurotransmitter (Loewi, 1921; Zimmer, 2006). Furthermore, by showing that physostigmine potentiated the actions of ACh, Loewi, and Navratil established that the heart contains an endogenous esterase for ACh, supporting the concept of a “cholinesterase” (Loewi and Navratil, 1926). In the next 15 years ACh was shown to serve as a neurotransmitter in autonomic ganglia and at the neuromuscular junction (Feldberg and Gaddum, 1934; Dale et al., 1936). The demonstration that ACh was released by simulation of motor nerves, that direct injection in muscle produced the kind of contraction elicited by nerve stimulation and that the response to ACh was increased by blocking cholinesterase activity strongly supported its role as a neurotransmitter for motor neurons (Feldberg and Gaddum, 1934; Brown et al., 1936; Bennett, 2000).

The case for ACh as a neurotransmitter in CNS built on insights from studies of its actions in the peripheral nervous system. Indeed, the use of drugs that increased the levels of ACh or blocked its actions played a key role in implicating ACh in memory. In memory studies on rodents, Deutsch and colleagues provided compelling evidence, demonstrating that effects of drugs that impact ACh levels are a function of the age of the memory, the dose of the drug, and the efficiency with which a task was initially learned (Deutsch, 1971; Squire, 1982). Importantly, while cholinergic neuronal systems were thus implicated in memory, the possible participation on non-cholinergic systems was considered possible. Studies in humans confirmed a role for cholinergic neurotransmission in memory. Those of Drachman and Levitt proved highly influential. Treatment with scopolamine, a competitive antagonist of acetylcholine at muscarinic receptors, induced in normal young human subjects impairment of memory storage, and retrieval as well as cognitive non-memory tasks with sparing of immediate memory (Drachman and Leavitt, 1974). The authors noted that the changes were strikingly similar to the changes seen in normal aged subjects (average age of ∼67 years) and speculated that deficits in cholinergic function might underlie age-related memory loss. That the latter was a common occurrence in humans and other mammals was widely appreciated by mid- to late 1970s, leading to the enunciation of the “cholinergic hypothesis of age-related memory dysfunction” and the consideration that such changes were present during both normal aging as well as, and to a greater extent in, AD (Bartus et al., 1982). An important development came from studies in AD brain noting selective reduction of the activity of the acetylcholine synthetic enzyme choline acetyltransferase (ChAT) in hippocampus, a region known to participate in memory functions, as well as in cortex and amygdala, with lesser reductions in other brain regions. A similar pattern of reduced activity was noted for acetylcholinesterase (AChE), but not for the activity of glutamic acid decarboxylase (GAD), the synthetic enzyme for g-aminobutyric acid (GABA). These data pointed to selective failure of the cholinergic system and raised the possibility that this change contributed to the pathogenesis of AD (Davies and Maloney, 1976). The reduction of ChAT activity was later confirmed and expanded to other brain regions of AD patients (Perry et al., 1977; Davies, 1979). Remarkably, when cases were stratified by age, a decrease in ChAT activity was apparent in older vs. younger controls, suggesting age-related changes in cholinergic function in asymptomatic aged people (Davies, 1979). A correlation between ChAT activity and mental test score in demented subjects suggested that deficits in ACh synthesis could underlie cognitive dysfunction (Perry et al., 1978).

With a role for cholinergic dysfunction posited for AD, the next step was to define the cholinergic neurons whose axons innervate cortex. The first documentation was provided by Johnston, McKinney, and Coyle in 1979 (Johnston et al., 1979). Studies in which kainic acid was used to selectively lesion neurons in the basal forebrain of rats resulted in striking reductions in ChAT, ACh, and the activity of synaptosomal high-affinity choline uptake, but not in neurochemical markers of GABAergic, noradrenergic, or serotoninergic neurons in ipsilateral neocortex. The locus of ChAT expressing neurons was defined as the nucleus basalis of Meynert (NBM) (Johnston et al., 1979) (see below for the anatomy of basal forebrain cholinergic neurons (BFCNs). With the locus of cortical cholinergic afferents now established, it remained to be described whether or not they were impacted in AD. This was evidenced by marked loss neurons of NBM neurons in the postmortem AD brain (Whitehouse et al., 1981, 1982). The role ascribed to hippocampus in memory pointed to cholinergic denervation of this region as likely contributing to memory loss, a suggestion consistent with evidence for marked neuron loss in the NBM in AD patient samples (Coyle et al., 1983).

Additional persuasive support for the “cholinergic hypothesis” came from clinical trials demonstrating efficacy for an AChE inhibitor in improving cognition in patients with AD. The initial effort, led by Summers and colleagues, evaluated tetrahydroaminoacridine (THA; Tacrine) treatment of subjects with AD using daily global assessment, the Names Learning Test, the Orientation Test, and the Alzheimer Deficit Scale. They showed improvement: (1) in an unblinded study (*n* = 17); and (2) in a blinded, placebo-controlled, crossover trial (*n* = 15 of the original group). Long term treatment (*n* = 12 of the original group) reported showing no serious side-affects and as providing continued symptomatic cognitive benefit of varying degree (Summers et al., 1986). The authors pointed to THA as a symptomatic, not curative, treatment and suggested that benefits would not be long-lasting. One follow-on study demonstrated statistically significant, dose-related improvements on clinician interview-based impression (CIBI), and the Alzheimer’s disease assessment scale-cognitive subscale (ADAS-Cog) (Knapp et al., 1994). But not all such studies confirmed benefits for cognition and behavior and concerns were raised due to evidence of hepatic dysfunction. Approved by the FDA in 1993, Tacrine was later withdrawn due to concern for hepatotoxicity (Anand and Singh, 2013). Nevertheless, robust efforts to build on the cholinergic hypothesis led to discovery and development of additional AChE inhibitors. Currently, three such agents are FDA-approved for treatment of AD-related cognitive dysfunction: donepezil, rivastigmine, and galantamine (Anand and Singh, 2013). Meta-analyses of their use demonstrate significant effects on improving cognition, typically as assessed using the ADAS-Cog, as well as clinician-rated global measures and measures of function and behavior. It is important to note that while such changes are significant, and long-term use is correlated with benefits for cognition and function, the benefits conferred by AChE inhibitors are modest (Deardorff et al., 2015).

Subsequent years have seen significant advances in understanding the anatomy of the basal forebrain cholinergic complex and the role BFCNs play in cognition. The cell bodies of BFCNs are found in a series of nuclei in the basal forebrain, including the medial septal nucleus (MSN), the diagonal band of Broca (DBB) (including both vertical and horizontal domains), the NBM, and the substantia innominate (SI) (Ballinger et al., 2016). In primates, the cholinergic nuclear groups are referred to as: Ch1 = MSN; Ch2 = vertical limb of the DBB; Ch3 = horizontal limb of the DBB; and Ch4 = the basal magnocellular complex that includes the SI, the NBM, the magnocellular preoptic nucleus and ventral pallidum (Mesulam et al., 1983). The axons emerging from these nuclei demonstrate distinct and characteristic pattern of innervation: the MSN and vertical limb of the DBB send axons to hippocampus, entorhinal cortex, and parahippocampus; the horizontal limb of the DBB, NBM, and SI send projections to neocortex and amygdala. A wealth of data now point to BFCNs, and their release of ACh, as supporting cognitive processes, including attention and memory (Ballinger et al., 2016). In earlier studies, selective lesions of BFCNs using saporin conjugated to an antibody against p75 neurotrophin receptor (p75NTR) (see below) impaired cognitive functions (Johnson et al., 2002; Conner et al., 2003; Knox, 2016). More recently, optogenetics has been used to explore BFCN contributions to learning and memory, demonstrating a role in a range of cognitive behaviors (Hangya et al., 2015). As expected, different portions of the forebrain cholinergic complex are implicated in different cognitive functions (Knox, 2016; Boskovic et al., 2018; Staib et al., 2018). Dysfunction and loss of BFCNs is now accepted as playing a significant role in cognitive dysfunction in AD (Ballinger et al., 2016).

The emergence and testing of the cholinergic hypothesis stands as an important milestone in the pursuit of the pathogenesis and treatment of AD. But it was evident that cholinergic hypothesis would not account fully for the pathophysiology of AD. Indeed, even as the cholinergic hypothesis emerged observations pointed to degeneration of other non-cholinergic populations in AD (Price et al., 1985). Nor did inhibition of AChE provide more than a modest benefit for cognition. Nevertheless the cholinergic hypothesis was highly influential. Some years later, Mesulam commented: “The cholinergic pathway emanating from the basal forebrain constitutes one of the most important modulatory afferents of the mammalian cortex. The initial expectation that the cholinergic deficiency would provide a unifying pathophysiological basis for Alzheimer’s disease and that cholinergic therapies would cure the dementia were clearly too optimistic. Nonetheless, the cortical cholinergic denervation remains one of the earliest, most severe, and most consistent transmitter changes in this disease. The cholinergic depletion may provide an important substrate for the neuropsychological features of Alzheimer’s disease, and may eventually yield important clues to its pathogenesis.” Indeed, this has proven to be the case as the involvement of BFCNs inspired studies of other approaches to pathogenesis. One such approach pursued a search for neurotrophic factors active on BFCNs to decipher whether deficient trophic support contributes to pathogenesis.

## Ngf-Cholinergic Neuron Axis

The selective vulnerability of neuronal populations in AD and AD-DS should serve as a source of insights into pathogenesis. The early, selective loss of cholinergic neurons is yet to be fully understood, but an early suggested a link to deficient neurotrophic support. As suggested by Appel, neurodegeneration may be due to failure of innervated cells, including hippocampal and cortical neurons, to supply a relevant cholinergic neurotrophic factor (NTF), resulting in functional impairment of MSN and NBM neurons (Appel, 1981). If a deficient supply of a NTF contributes to pathogenesis it is rational to propose that treatment with an exogenous source of the NTF would restore the deficit and protect neurons from dysfunction and death. At the time of the Appel hypothesis, which might be called “the neurotrophic factor deficiency hypothesis,” relatively few NTFs had been identified and none were known to be active on CNS neurons. The first and best characterized was nerve growth factor (NGF) a small polypeptide discovered in the early 1950s by Levi-Montalcini and Hamburger with robust effects on sensory and sympathetic neurons (Levi-Montalcini and Hamburger, 1951). The first convincing suggestion that NGF might act in the brain was the demonstration by Seiler and Schwab that injecting radiolabeled NGF in the cortex of the rat was followed by selective retrograde transport to the basal forebrain cholinergic complex (Seiler and Schwab, 1984). This suggested that BFCNs expressed a receptor for NGF and may be responsive to NGF. Though the significance of this finding was little appreciated at the time it inspired studies over the next decade that provided a convincing demonstration that NGF does serve as a target-derived NTF for BFCNs. The key elements of such a demonstration include the following: (1) that the factor is produced in the target of innervation; (2) that it is released therein to bind to specific receptors on innervating axons; (3) that receptor-mediated signaling is registered in receptive neurons; and (4) that signaling events are linked to differentiation and/or maintenance of these neurons. Each of these criteria were satisfied for NGF and BFCNs. Critical steps in this line of investigation began with demonstrating that NGF acts on BFCNs and later to the demonstration of its expression in target territories and the expression of its receptors on BFCNs. Thus, the following was shown: (1) *in vitro* and *in vivo*, NGF acted in a dose-dependent fashion on the neurochemical differentiation of cholinergic neurons of the basal forebrain by significantly increasing the expression of activity of ChAT (Gnahn et al., 1983; Hefti et al., 1985; Mobley et al., 1985, 1986; Hagg et al., 1989; Li et al., 1995); (2) NGF intracerebroventricular (ICV) injection resulted in sparing of BFCNs in several lesion models, as measured by neuron size and number (Hefti, 1986; Williams et al., 1986; Kromer, 1987; Figueiredo et al., 1996); (3) NGF treatment reversed atrophy of BFCNs in a mouse model of DS (Holtzman et al., 1993); (4) NGF mRNA and protein were demonstrated in the target regions of BFCNs in hippocampus and cortex, with greater *NGF* expression in interneurons (Korsching et al., 1985; Large et al., 1986; Shelton and Reichardt, 1986; Whittemore et al., 1986; Ayer-Lelievre et al., 1988; Rocamora et al., 1996); (5) NGF present in target territories was selectively internalized and retrogradely transported in the axons of BFCNs (Seiler and Schwab, 1984); (6) cholinergic denervation of hippocampus induced a transient accumulation of NGF in hippocampus without a change in NGF mRNA (Korsching et al., 1986); (7) in developing rodents, disrupting *NGF* gene expression, or antibody-mediated sequestration of NGF, decreased expression of ChAT, and reduced the developmental increase in BFCN size and number (Crowley et al., 1994; Li et al., 1995; Debeir et al., 1999).

An interesting feature of the NGF-BFCN connection was evidence not just of the continued presence of NGF in target territories of mature BFCNs but that NGF acting on mature neurons robustly regulates trophic status. For example, prolonged infusion of NGF in adult mice deleted for one copy of *NGF* (i.e., NGF^+/-^ mice) increased the cell size of BFCNs and the density of cholinergic innervation in hippocampus (Chen et al., 1997). In another example, in the elderly Ts65Dn mouse model of DS, in which there was reduction in the size and number of these neurons as well as decreased in cholinergic innervation of hippocampus, intraventricular delivery of NGF restored each of these measures to normal (Cooper et al., 2001). The latter findings were attributed to the ability of NGF to reverse the “phenotypic silence” of BFCNs – i.e., the downregulation of markers of BFCN differentiation in the setting of deficient supply of NGF signaling (Cooper et al., 2001). Moreover, NGF treatment of the wild type controls of aged Ts65Dn mice also increased the density of their cholinergic fibers in hippocampus, suggesting that augmenting normal levels of NGF serves to increase trophic status in normal neurons (Cooper et al., 2001). Continued NGF signaling in BFCNs may point to a role for NGF in dynamically modulating the synaptic function in hippocampal and cortical circuits. The robust responsiveness of BFCNs to increasing NGF levels mirrors a much earlier demonstration of the same phenomenon in sympathetic neurons in which it was speculated that endogenous NGF acting on adult neurons plays a regulatory role directed at maintenance of the adrenergic terminal network (Bjerre et al., 1975).

As work on NGF progressed a critical task was to identify the receptor(s) that mediated NGF signaling. Two receptors were discovered. The first was p75NTR (Johnson et al., 1986; Radeke et al., 1987). p75NTR is a transmembrane glycoprotein that binds with approximately nanomolar affinity to all members of the neurotrophin family, of which NGF is a member together with BDNF (brain-derived neurotrophic factor), NT3 (neurotrophin 3), and NT4 (Sofroniew et al., 2001; Chao, 2003). p75NTR also binds to the pro-forms of NGF and other neurotrophins with subnanomolar affinity. A member of the death domain-containing receptor subgroup of the tumor necrosis factor (TNF) receptor family, p75NTR signaling appears to serve a number of functions. Its signaling is cell context-specific, owing to the existence of co-receptors, signaling adaptors, and possibly other cell-specific features. p75NTR signaling shares features with other death receptors, including signaling through NF-κB and c-Jun N-terminal kinase (JNK) pathways as well as through RhoA. In perhaps the best studied neuronal population, p75NTR signaling in sympathetic neurons promotes axonal degeneration and cell death (Bothwell, 2016). Moreover, it appears that p75NTR regulates pruning of BFCN axonal arbors. Cre-mediated knockout of p75NTR in BFCNs significantly increased synaptic connectivity in medial prefrontal cortex, an effect that was prevented by co-expressing the p75NTR intracellular domain in these neurons. Thus, p75NTR appears to control BFCN synaptic arborization (Boskovic et al., 2018) and may do so in the mature CNS. What role(s) are played by retrograde axonal transport of p75NTR and its signaling properties in BFCNs is unknown, but recent data point to transport of the intracellular domain of p75NTR in mediating pro-apoptotic signaling in sympathetic neurons (Pathak et al., 2018). Consistent with these data, and in agreement with earlier studies pointing to increased differentiation and numbers of BFCNs in p75NTR knockout mice, a recent study showed that selective loss of p75NTR in BFCNs resulted in reduced cell death during the early postnatal period (Yeo et al., 1997; Naumann et al., 2002; Boskovic et al., 2014).

Another p75NTR function is to positively modulate trophic signaling through tropomyosin receptor kinase A (TrkA) (Davies et al., 1993; Bibel et al., 1999), the second NGF receptor discovered (Kaplan et al., 1991). TrkA, a member of the Trk receptor family which also includes TrkB (a receptor for BDNF and NT-4), and TrkC (a receptor for NT-3 receptor) (Huang and Reichardt, 2001; Chao, 2003) is activated by NGF alone among the neurotrophins (Belliveau et al., 1997). TrkA is a receptor tyrosine kinase whose activation by NGF leads to dimerization with tyrosine auto-phosphorylation resulting in the activation of classical signaling cascades of the mitogen-activated protein kinases (MAPK), phosphoinositide 3-kinase (PI3K), and phospholipase C-γ (PLC-γ) pathways (Bothwell, 2016). TrkA activation is responsible for the classical trophic effects of NGF on BFCNs. TrkA knockout mice show a marked decrease in the number of BFCNs and decreased cholinergic fiber density in hippocampus, indicating that NGF signaling through TrkA is essential for the normal development of BFCNs (Fagan et al., 1997). Enabling target-derived trophic support for BFCNs, retrograde axonal transport of activated TrkA delivers trophic signals to BFCN cell bodies (Xu et al., 2016). Both p75NTR and TrkA are robustly expressed in BFCNs and both are present on their axons (Holtzman et al., 1992, Kordower and Mufson, 1993; Holtzman et al., 1995; Fagan et al., 1997). Remarkably, as one facet of the response to NGF, expression of both TrkA and p75NTR were induced by NGF treatment *in vivo* (Miller et al., 1994; Li et al., 1995), pointing to the possibility that NGF signaling positively reinforces later signaling events.

Additional studies are needed to address the specific roles for p75NTR and TrkA in the development and maintenance of BFCNs and to address possible interactions between these receptors. In this context it is noteworthy that increased levels of pro-NGF were present in the AD and the adult DS brain, evidently due to reduced processing to the mature form of NGF. This raises the possibility that increased levels of pro-NGF, acting through p75NTR in BFCN axons, could compromise the integrity of axonal arbors, and synaptic function in AD (Bruno et al., 2009; Iulita and Cuello, 2014; Iulita et al., 2014). Moreover, p75NTR has been identified as one of the receptors for Aβ (Yaar et al., 1997; Yaar et al., 2002); robust p75NTR expression in BFCNs could thereby predispose them to Aβ-mediated signaling and endocytosis. Finally, speaking to the importance of continued NGF signaling in mature BFCNs, viral-mediated suppression of TrkA receptor expression in BFCNs acted selectively in aged rats to compromise attentional performance, reduce ACh release, and reduce ChAT immunostaining in BFCN cell bodies and cortical axons (Parikh et al., 2013). Taken together, the data are compelling that NGF and its receptors play critical roles in the development and maintenance of BFCNs and that studies of deficits in NGF and NGF signaling are germane to discussions of AD pathogenesis.

## Ngf/Trk Signaling From Endosomes; the Signaling Endosome Hypothesis

For target-derived NTFs to impact the function of neurons they must communicate signals over the long distances between the ends of axons and the corresponding neuron cell bodies. The most well studied means by which this occurs begins with endocytosis of the NTF/receptor complex. The NTF/receptor complex first identified as playing a role in long distance signal transmission consisted of NGF bound to its activated TrkA receptor. Endocytosis of the NGF/activated TrkA complex was shown to form the “signaling endosome,” an organelle that can then be retrogradely transported to the soma via dynein-based transport (Howe and Mobley, 2005; Wu et al., 2009; Harrington and Ginty, 2013; Chen et al., 2018). Remarkably, and apropos its name, the NGF/TrkA signaling endosome contains not only the internalized NGF/active TrkA complex but also on its cytosolic surface the activated isoforms of many signaling molecules involved in the MAPK, Akt and PLCγ pathways (Howe et al., 2001; Delcroix et al., 2003; Zweifel et al., 2005; Chen et al., 2018). Excellent reviews speak to the formation, composition and regulation of signaling endosomes (Harrington and Ginty, 2013; Cosker and Segal, 2014; Scott-Solomon and Kuruvilla, 2018).

Studies *in vivo* and *in vitro* showed that Rab5-positive early endosomes were the first endocytic compartment in the retrograde pathway for transmitting the NGF/TrkA signaling endosomes (Wu et al., 2001; Delcroix et al., 2003; Cui et al., 2007; Liu et al., 2007). Recent studies point to the participation of other endocytic compartment(s) in this process and raise the possibility of neuron-specific differences in cargo management (Saxena et al., 2005; Deinhardt et al., 2006; Chen et al., 2018; Ye et al., 2018). It follows that retrograde signaling of NGF is impacted by factors that regulate the endocytic pathway and that dysregulation of such factors could disrupt the ability of signaling endosomes to support the long-distance communication needed to ensure normal neuronal structure and function. Rab5 belongs to the Ras superfamily of small Rab GTPases. Active Rab5 localizes to both the plasma membrane and early endosomes. Through its localization it is poised to regulate endocytosis of surface proteins and to regulate their trafficking through the early endosomal compartment (Zerial and Mcbride, 2001). It is noteworthy that AD risk variants include several genes whose proteins regulate endocytosis and transport, among which are Bin1, and Rin3 (Karch and Goate, 2015).

## Ngf Signaling Dysregulation in Ad and Ad-Ds

The Appel hypothesis, pointing to a deficiency of NTF support as contributing to neurodegeneration, suggested that changes in the expression and release of such factors would be responsible. Given the robust harvest of data since that time, it is now possible to think about several levels at which an NTF deficiency might arise: NTF synthesis, NTF release, NTF binding to receptors, endocytosis of the NTF and its activated receptors into signaling endosomes, retrograde transport of signaling endosomes, and the robustness of signaling events catalyzed by signaling endosomes in the neuronal soma. There are data for many of these aspects for NGF and BFCNs in AD. NGF mRNA levels were not reduced in AD cortex or hippocampus in comparison to controls (Phillips et al., 1991; Jette et al., 1994; Fahnestock et al., 1996; Hock et al., 1998). In contrast, NGF protein levels were found to be increased in cortex and hippocampus (Crutcher et al., 1993; Scott et al., 1995; Fahnestock et al., 1996; Hock et al., 2000). Suggesting that decreased retrograde NGF transport contributed to this finding was that in the same brains increased NGF levels in cortex contrasted with decreased NGF levels in the cholinergic basal forebrain NBM (Scott et al., 1995). Supporting this interpretation, in the Ts65Dn mouse model of DS NGF transport from hippocampus to the basal forebrain was shown to be deficient, a change correlated with increased NGF in hippocampus and a trend to decreased levels in the MSN of the basal forebrain (Cooper et al., 2001). In contrast to the findings for NGF in the AD brain, BDNF protein was reduced in hippocampus and cortex as were BDNF mRNA levels (Siegel and Chauhan, 2000) suggesting a decrease in local synthesis for this NTF. There is little data for the temporal characteristics of NGF release in the targets of BFCNs, but one report speaks to long-lived increases in cortex following stimulation of the NBM (Hotta et al., 2007), suggesting that deficient innervation of cortex could reduce release.

Unlike NGF, there were decreases in the levels of both the mRNA and protein levels for TrkA in AD. Indeed, in surviving BFCNs in the NBM there was decreased TrkA mRNA (Boissiere et al., 1997; Ginsberg et al., 2006); the decreases were correlated with decreases in mini-mental state examination (MMSE) scores (Ginsberg et al., 2006). Reductions in mRNAs for TrkB and TrkC were also evident (Ginsberg et al., 2006). Corresponding to the reduction in mRNA, TrkA protein levels were also decreased in cell bodies of BFCNs as well as in AD cortex (Mufson et al., 1997). TrkA protein levels were also reduced in surviving neurons in NBM in DS (average age ∼50) (Sendera et al., 2000). In contrast to the findings for TrkA there was no change in the mRNA for p75NTR in basal forebrain, including the NBM (Goedert et al., 1989; Ginsberg et al., 2006). However, in one report remaining neurons and their processes were less immunoreactive for p75NTR (Salehi et al., 2000).

The studies just reviewed are evidence against a change in NGF gene expression as responsible for degeneration of BFCNs. The reduction in TrkA raises the possibility that this could contribute, but given evidence for decreased NGF retrograde transport and studies showing that NGF positively regulates TrkA gene expression, reduced retrograde NGF signaling could explain the decrease in TrkA gene expression. The question then is whether reduced retrograde transport of NGF signaling contributes to the degeneration of BFCNs in AD. This question has been addressed in a model of DS, the Ts65Dn mouse, whose genome is segmentally trisomic for approximately 100 mouse orthologs on human chromosome 21. The mouse App gene is present in three copies. *In vivo* increased *App* dose caused endosomal defects in retrograde trafficking of NGF in BFCNs in these mice (Salehi et al., 2006). This was manifest in BFCN axons in hippocampus by increased immunostaining for APP and its CTFs in enlarged Rab5-positive endosomes; thus, endosomes containing NGF and its receptors were significantly larger in cholinergic axons in Ts65Dn vs. control (2N) mice. Correspondingly, transport of NGF from hippocampus to basal forebrain was reduced to ∼10% of 2N controls (Salehi et al., 2006); synaptosomal studies showed that decreased transport was not due decreased NGF binding or internalization (Cooper et al., 2001). The defect in transport was largely restored by normalizing *App* dose. Significantly, reducing App dose also prevented the degeneration of BFCNs in the Ts65Dn mouse, pointing to defective retrograde signaling of NGF as responsible for BFCN loss (Salehi et al., 2006). Using the same methods to examine NGF transport, mice that overexpressed wild type human *APP* or a mutant of human *APP*, *APP*_Swe_ also showed a modest but significant reduction in NGF transport (Salehi et al., 2006). That App overexpressing mice demonstrate a more general disruption of endosomal function is evidence that transport of BDNF signaling was also deficient in the Ts65Dn cortex. Thus, enlarged Rab5-positive endosomes containing activated TrkB receptors were seen in synapses and there was a corresponding decrease in TrkB-positive endosomes in cell bodies (Nosheny et al., 2015).

To explore the molecular basis by which increased APP gene dose compromises NGF retrograde transport studies were carried out on rat BFCNs *in vitro*. Overexpression of APP or C99, in BFCNs each induced increased activation of Rab5 and enlargement of early endosomes. These changes were correlated with decreased retrograde axonal transport of NGF, decreased NGF signaling, and atrophy of BFCNs (Jiang et al., 2010; Kim et al., 2016; Xu et al., 2016). Significantly, C99 mediated atrophy was mediated by increased activation of Rab5 because it was prevented when a dominant negative version of Rab5 was co-expressed (Xu et al., 2016). These data are evidence that increased APP gene dose, as reflected in increased levels of its products, including C99, acts to increase activation of Rab5 and reduce retrograde transport of NGF and NGF signaling in BFCNs. These findings may be directly relevant to dysfunction and loss of BFCNs in both AD and AD-DS.

## Explaining the Selective Vulnerability of Bfcns: a Role for Deficient Ngf Signaling

**FIGURE 1:**
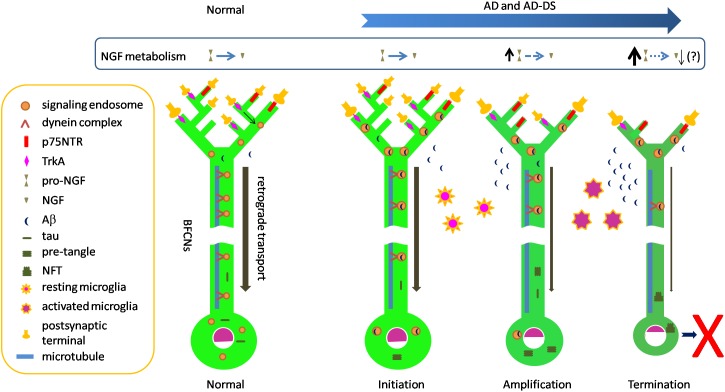
Speculative scheme to explain selective vulnerability of BFCNs in AD and AD-DS. Normal: dynein-mediated retrograde transport provides a steady flow of NGF/TrkA-containing Rab5-positive signaling endosomes from synapses to cell bodies. In this way target-derived NGF released by target neurons in hippocampus and cortex dynamically regulate the structure and function of BFCNs. The remarkable length of BFCN axons and their widespread arborization in target fields creates the need to move trophic signals over long distances. Initiation: Aβ42 and Aβ oligomeric species accumulate in BFCNs. Together with increased levels of C99 these species may contribute to the dysregulation of early endosomes seen at this stage. Dysregulation of endosomes reduces axonal transport of NGF/TrkA signaling endosomes, thus compromising trophic support of BFCNs. p75NTR binds and internalizes Aβ. Tau in pre-tangles is present, possibly reflecting increasing levels of tau oligomers which may also impact axonal transport. Meanwhile, resting microglial cells respond to Aβ and become activated and migrate to plaques to phagocytose Aβ. Amplification**:** Intracellular Aβ continues to impact BFCNs and failing retrograde transport of NGF signals results in BFCN dysfunction as measured by reduced expression of the genes for TrkA and ChAT. Reduced synthesis and anterograde transport of proteins needed for synaptic function and maintenance of axonal arbors reduces synapse number and function. p75NTR gene expression is relatively maintained, creating an imbalance in NGF signaling with excessive pruning of axonal arbors. Deficient activation of BFCN-responsive postsynaptic neurons may result in reduced processing of pro-NGF to mature NGF. Increased pro-NGF may increase p75NTR signaling. Termination: continuing compromise of retrograde axonal transport of NGF/TrkA signaling endosomes severely compromises the trophic status of BFCNs with marked changes in cell bodies and further shrinkage of axonal arbors and synaptic dysfunction. Sustained microglial activation results in defective Aβ phagocytosis and release of proinflammatory cytokines. The cumulative effect of these changes is atrophy and eventual death of BFCNs.

Selective vulnerability of neurons is a well appreciated but poorly understood feature of many neurodegenerative disorders. As discussed above, selective loss of BFCNs characterizes AD. This question arises as to why BFCNs are more vulnerable than many other populations. Amyloid and tau pathologies, including NFTs and p-tau in pre-tangles, impact BFCNs relatively early in AD. It is likely that these markers significantly postdate the presence and toxic actions of oligomers of Aβ and tau. Such species are likely to have direct access to BFCNs. Addressing this suggestion, one can ask how oligomers of Aβ and tau might predelict BFCNs for degeneration. Below we speculate a series of events by which the biology of BFCNs and their dependence on NGF may feature in selective vulnerability. The stages of degeneration are assigned to distinct, but likely overlapping, temporal phases: initiation, amplification and termination (Figure 1).

### Initiation

What events might initiate the process of BFCN degeneration? Increasing levels of soluble oligomeric Aβ42 species in BFCNs may represent the earliest molecular provocation. Absolute increases in Aβ42 are present in DS and FAD due to APP gene triplication. Pathogenic mutations in PSEN and APP converge on impairment of the sequential cleavage efficiency, leading to production of longer and more hydrophobic Aβ peptides (Szaruga et al., 2017). Indeed, due to γ-secretase dysfunction, reduced clearance of longer and more hydrophobic Aβ peptides, or both, Aβ42 accumulates in both FAD and SAD (Selkoe and Hardy, 2016). Focusing on BFCNs, Geula and colleagues showed that intraneuronal immunopositivity for Aβ42, including the amino terminus of Aβ, was present in these neurons in young brains (ages 20–66) as well as in older non-demented and AD cases. There was no quantitative difference between groups. Staining with one Aβ42 antibody revealed granular, punctate staining consistent with the presence of Aβ42 within a vesicular compartment. Virtually all BFCNs were immunopositive at all ages and immunoreactivity in BFCNs exceeded that in cortical regions and in another magnocellular group in globus pallidus. Interestingly, BFCN homogenates demonstrated oligomeric Aβ species of a variety of sizes, with increased levels of a cluster of bands between ∼35 and 44 kDa in non-demented elderly, and to a greater extent, in AD, in the absence of increases in either APP or total Aβ (Baker-Nigh et al., 2015). The presence of Aβ within early endosomes may be especially relevant to initiation of pathogenesis. Aβ is present in early endosomes in early stage AD and in the young DS brain. The finding that early endosomal enlargement is one of the earliest pathological manifestations of AD and AD in DS, raises the possibility that Aβ within endosomes acts to induce hyperactivation of Rab5 with dysregulation of this compartment and endosomal enlargement through increased fusion of early endosomes. Whether or not and how Aβ within endosomes induces activation of Rab5 is unknown, but if Aβ impacts processing of APP within endosomes, for example by compromising the activity of γ-secretase, this would lead to increased levels of C99. Importantly, C99 levels are increased in FAD and, more variably, in SAD (Pera et al., 2013). Due to increased APP dose C99 levels are also increased in the DS brain. With disruption of endosome size comes a reduction in endosomal trafficking, as has been demonstrated (Xu et al., 2016). While a variety of cargoes would be impacted, in BFCNs this would prominently impact retrograde transport of NGF signaling endosomes and thus compromise their trophic support of BFCNs, including the genetic and cellular programs that support structure and function of these neurons (Figure 1). Quite possibly this stage also features interaction of Aβ-mediated and tau-mediated toxic events; the latter may include compromised transport of TrkA (Vossel et al., 2010, 2015). Aβ-mediated dysregulation of tau homeostasis, which has been demonstrated (Zheng et al., 2002; Stoothoff and Johnson, 2005), could initiate increased levels of p-tau and tau oligomer formation. In this context it is noteworthy that tangle and p-tau pre-tangle cytopathology and immunoreactivity were present in the NBM in aged normals as well as in those with MCI, with increased frequency in the latter. Remarkably, in cognitively unimpaired subjects at the earliest stages of tau pathology (i.e., Braak Stage 1 and 2), apart from the medial temporal lobe, the NBM was the only forebrain structure to show tau cytopathology, pointing to an unusual propensity to tau accumulation in these cells (Mesulam et al., 2004). In this early AD stage microglia would respond to Aβ by becoming activated and migrating to plaques to phagocytose and clear Aβ (Kinney et al., 2018).

### Amplification

What elements of BFCNs would amplify the effect of increasing levels of toxic Aβ and tau oligomers on pathogenesis? Several possibilities can be envisioned. First, the need for BFCNs to engage in both volume-based and point-to-point synaptic neurotransmission may impose considerable metabolic demands (Ballinger et al., 2016). Second, there is need for continued NGF signaling to maintain the differentiated status of BFCNs (Counts and Mufson, 2005). Third, the remarkable length of BFCN axons and their widespread arborization in target fields creates the need to move trophic signals over very long distances in relatively thin unmyelinated axons. A recent study notes that a single human BFCN may be ∼100 m long and that the highly arborized distal axon, with perhaps more than a 1000 branch points per arbor, covers a very large volume of cortex. Added to this, the arbors converge on a single relatively long (10 cm) proximal axon whose diameter is ∼0.3 μm (Wu et al., 2014). Fourth, given complex axonal arbors, as additional burden may attend trafficking of NGF signals within enlarged, abnormal endosomes. Fifth, the presence on BFCN axons of p75NTR may serve to bind and internalize Aβ (Yaar et al., 2002) and possibly AβOs. Indeed, there is evidence that soluble and aggregated Aβ bind to p75NTR (Yaar et al., 1997). Sixth, with failed retrograde transport of NGF signals there would be reduced expression of TrkA receptors, as has been demonstrated (Mufson et al., 1997), further compromising the ability to respond to NGF present in target territories. Given that p75NTR gene expression appears to be less severely affected (Ginsberg et al., 2006), persistence of p75NTR on BFCN axons may negatively impact axonal arbors, possibly through pruning (Schuldiner and Yaron, 2015). One consequence of deficient signaling to postsynaptic neurons could be reduced ability to properly process NGF in cholino-receptive target neurons, leading to the presence of increased levels of pro-NGF, a ligand for p75NTR that could signal to further disrupt axonal arbors and synaptic function. The progressive decrease in ChAT activity characteristic of later stages in AD (Davis et al., 1999) could be explained by increasing failure to transport NGF signaling to the cell bodies of BFCNs (Figure 1).

### Termination

The events just outlined would lead to a significant and increasingly severe compromise of the ability of BFCNs to maintain key facets of its differentiated state with downregulation of genetic and cellular programs that support maintenance of and synaptic signaling from axonal arbors. Another feature at this stage of disease could be sustained activation of microglial cells, which might exacerbate AD through a combination of defective Aβ phagocytosis and release of proinflammatory cytokines (Kinney et al., 2018). Activated microglia could contribute to neuronal injury. The cumulative effect of these changes would be atrophy and eventual loss of BFCNs. Thus, the process leading to death of these cells would proceed over decades – from early events triggered by toxic Aβ and tau oligomers, to progressive compromise of retrograde NGF signaling, to increasing deficits in synaptic function, to dysregulation of secretion of NGF from postsynaptic targets and activation of nearby microglia, to eventual demise of BFCNs (Figure 1).

## Therapeutic Strategies in Ad and Ad-Ds

Given its trophic role for BFCNs, NGF has been considered a potential therapy to prevent loss of these neurons in AD, and DS. An important question is how to deliver NGF. Due to its size, NGF does not cross the blood-brain barrier when administered peripherally (Granholm et al., 1998), thus precluding this method. ICV NGF administration has been practiced effectively in rodent models and was delivered in this fashion in a pilot clinical trial. Treatment of three patients was accompanied by constant back pain and weight reduction (Eriksdotter Jonhagen et al., 1998). Pointing to another possible complication, continuous ICV delivery of NGF in the rat resulted in marked, albeit reversible, Schwann cell hyperplasia, possibly in response to sprouting of NGF-responsive sensory and sympathetic neurites at the dorsolateral caudal medulla and upper cervical spinal cord (Winkler et al., 1997). Studies in primates also demonstrated Schwann cell hyperplasia (Day-Lollini et al., 1997). To avoid these complications an alternative method for NGF delivery, allowing for local delivery to the basal forebrain, was employed by Tuszynski and colleagues (Tuszynski et al., 2005). NGF gene therapy was effected via introduction into the basal forebrain of autologous fibroblasts induced to express NGF or via an NGF-expressing adeno-associated viral vector (serotype 2) (AAV2). Classic trophic responses were detected in the basal forebrain. Increased levels of NGF were present in basal forebrain up to one year after viral delivery. Patients did not report pain and there was no evidence of Schwann cell hyperplasia (Tuszynski et al., 2015). A Phase Ib study of NGF gene therapy used AAV2-mediated delivery to the basal forebrain bilaterally in 10 AD patients. AAV2-NGF was reportedly safe and well tolerated. At autopsy, performed in 5 patients up to 6 years following delivery of NGF-encoding virus, NGF immunostaining was present near the needle tract and nearby BFCNs stained positive for NGF, p75NTR, and ChAT. There was also evidence of increased size of BFCNs near the site of injection. Cognitive measures showed no change, but sample size was small (Rafii et al., 2014). In a recently reported Phase II trial, 49 subjects with mild to moderate AD were randomized to receive stereotactically guided injection of AAV2-NGF or sham surgery. AAV2-NGF therapy was safe and well tolerated but there was no significant difference in either the primary or secondary outcome measures for cognition or function (Rafii et al., 2018). In yet another approach to local delivery of NGF to basal forebrain, a recent study reports the neurosurgical introduction of a device encapsulating NGF secreting cells; two devices were implanted in each hemisphere. Four patients with mild to moderate AD were examined for 6 months in an open-label Phase Ib study; the treatment was well tolerated and there was evidence for continued secretion at the time the devices were retrieved (Eyjolfsdottir et al., 2016). While the rationale for CNS delivery of NGF-based treatments is clear, effecting this form of treatment entails invasive measures.

An alternative to NGF itself would be delivery of a small molecule (Bruno et al., 2004) or monoclonal antibody agonist of TrkA (Lesauteur et al., 1996). Use of these modalities would facilitate delivery to axons, rather than the cell bodies, of BFCNs and would thus more closely address the locus of NGF deficiency in AD. Having stated this, one still has to address the concern that disrupted transport of NGF or NGF signaling may compromise delivery to BFCN cell bodies. The possibility that treatment efficacy may require higher cortical and hippocampal levels of NGF or NGF-mimics is suggested by deficient trophic responses in cultured BFCNs in which C99 overexpression was used to induce hyperactivation of Rab5 resulting in reduced retrograde NGF transport. Under these conditions, BFCN soma size in C99 expressing neurons was far less responsive than controls even when their axons were bathed in much higher than physiological levels of NGF (Xu et al., 2016). Finally, a small molecule antagonist of p75NTR (LM11A-31-BHS) aimed at improving synaptic function is in a Phase II trial (NCT03069014).

Addressing more generally the use of treatments directed at the pathogenesis of AD and AD-DS, and in view of increased APP gene dose in DS as well as changes in the processing of APP with absolute or relative increases in Aβ42 in the brain in all forms of AD, a focus on reducing the levels of APP and its products is rational. In DS, reducing the levels of APP mRNA and protein may prove effective and could be delivered well before the onset of AD pathogenesis. One potential strategy for DS is the use of RNA-based treatments [e.g., antisense oligonucleotides (ASOs)] to reduce APP mRNA levels (Sawa et al., 2018). ASOs have been reported to modestly lower APP protein in AD mouse models (Kumar et al., 2000; Erickson et al., 2012; Farr et al., 2014). Given restricted CNS access of peripherally administered ASOs, intrathecal delivery of ASOs may be required; this mode of delivery has recently been shown to be effective in treating neurodegenerative disorders (Schoch and Miller, 2017). Another RNA-based approach is suggested by a study using nanoparticles containing siRNAs against APP (Shyam et al., 2015). Alternatively, small molecules that interfere with translation of APP mRNA may prove useful. Posiphen is one such molecule; it reduced the levels of APP and its products, including Aβ42, in a translation-dependent manner in AD mouse models (Lahiri et al., 2007; Teich et al., 2018). In studies on a small number of MCI patients, treatment with Posiphen for 10 days reduced in CSF the secreted amino terminal fragments of APP (i.e., sAPPα and sAPPβ), total tau, p-tau, and showed a trend toward reduced Aβ42 (Maccecchini et al., 2012). Posiphen was also shown to reduce App protein in the Ts65Dn mouse model of DS (Salehi et al., 2008).

At present the pipeline of AD treatments lists 112 different agents, of which 63% aim at disease modification, 22% address cognitive symptoms, and 12% focus on psychiatric and behavioral symptoms (Cummings et al., 2018). To date there is no convincing evidence of efficacy for disease-modifying approaches, many of which have targeted APP processing (Cummings et al., 2018). Inhibiting the synthesis of Aβ through use β-secretase inhibitors generated a great deal of interest resulting in development and clinical testing of several compounds. Recent Phase III trials of three such drugs (Verubecestat, Atabecestat, and Lanabecestat), examining patient cohorts ranging from those at risk for AD to those with MCI, demonstrated not only lack of efficacy but evidence of worsened cognition (Panza et al., 2019). Equally disappointing were studies of γ-secretase inhibitors, including Begacestat, Semagacestat, and Avagacestat. Clinical trials showed lack of efficacy and worsened cognition (Panza et al., 2019). A much more promising rationale focuses on enhancing the processing of APP to reduce the levels of Aβ42. Several well-tolerated small molecule modulators of γ-secretases (GSMs), which act to enhance processivity of the enzyme complex, significantly reduced Aβ42 as well as Aβ40 *in vitro* and *in vivo* and reduced amyloid burden in mouse models of AD (Kounnas et al., 2010; Wagner et al., 2017). The possibility that GSMs impact both the endopeptidase as well as the exopeptidase (i.e., carboxypeptidase-like) activity of γ-secretases suggests that GSMs might also reduce the levels of C99, a topic that deserves additional study to counter the C99-mediated increase in activation of Rab5 and endosomal dysfunction.

There is a great deal of interest in the immune approach to Aβ through vaccination and passive immunization. These efforts followed considerable success using the immune approach in animal models of AD (Schenk et al., 1999; Das et al., 2001; Spencer and Masliah, 2014). An early vaccine trial was complicated by sub-acute meningoencephalitis (Hock et al., 2003; Orgogozo et al., 2003). This led to changes in vaccine design, including use of the N-terminus of the Aβ peptide and different adjuvants or display platforms (Muhs et al., 2007; Van Dyck, 2018). Several vaccine trials are ongoing (Castro et al., 2017). Considerable effort has focused on the use of monoclonal antibodies against Aβ. No fewer than seven such antibodies have been evaluated; clinical trials continue for solanezumab, gantenerumab, crenezumab, aducanumab, and BAN2401. The different epitopes used to generate antibodies, and the different Aβ species targeted, are noteworthy. The lack of understanding of which Aβ species, and in particular which toxic species, should be targeted, may limit success using the immune approach. Indeed, given data for a role for Aβ oligomers in AD pathogenesis it is noteworthy that at least 2 of the antibodies currently being evaluated are stated to not interact with oligomers (Van Dyck, 2018). In spite of the excitement generated by these trials, immune-based trials are yet to demonstrate efficacy. Nevertheless, the hope remains that antibody and vaccine approaches will show promise.

To achieve therapeutic efficacy without compromising neuronal function immune-based approaches may need to be informed by a detailed evaluation of all the Aβ targets addressed by antibodies. Indeed, Aβ monomers may be important for normal neuronal function. Aβ1-42 monomers were shown to be critical in maintaining neuronal survival and glucose homeostasis (Giuffrida et al., 2009, 2015). It has been suggested that both the gain of toxicity of Aβ oligomers and loss of the normal physiological function of the Aβ monomer may contribute to the pathogenesis of AD (Copani, 2017). Aducanumab, a human monoclonal antibody that selectively targets aggregated Aβ without binding to soluble Aβ monomers, may represent an agent that selectively targets toxic species (Sevigny et al., 2016). Unfortunately, two Phase III trials of Aducanumab in AD were recently halted due to the conclusion by an independent data monitoring committee that the trials were unlikely to meet their primary endpoints, which included measures of cognition (Servick, 2019). Use of advanced trial designs and the conduct of trials in presymptomatic or early symptomatic AD may support further developments. Equally or more important may be immune approaches informed by defining the structure of toxic Aβ conformers. Given the shared Aβ-related pathology between AD and DS, successful trials of vaccines and monoclonal antibodies in AD trials would support their use in DS. A recent study of vaccine targeting Aβ showed relative preservation of BFCNs in Ts65Dn mice without obvious side effects (Belichenko et al., 2016). A vaccine trial is underway in adults with DS (NCT02738450).

Together with increased interest in a role for tau in AD pathogenesis, efforts have also been directed to tau-directed therapeutics. Diverse approaches have been suggested (Holtzman et al., 2016). Among them, multiple tau-based immunotherapy strategies have been successfully tested in AD animal models, pointing to this as an option for treating AD (Asuni et al., 2007; Boutajangout et al., 2011; Pedersen and Sigurdsson, 2015). Several tau-based immunotherapies have entered clinical trials in AD. Active immunotherapies, including AADvac-1 and ACI-35, target misfolded tau or p-tau epitopes in Phase II and Ib trials, respectively. Anti-tau antibodies, including ABBV-8E12 and RG6100, are in Phase II trials (Medina, 2018). As for Aβ, successfully targeting tau may benefit from defining the structure of toxic tau species. Future trials may also explore other facets of tau pathology, including the role that DYRK1A plays in priming tau for phosphorylation by other kinases (Wegiel et al., 2011).

The hyperactivation of Rab5 resulting in early endosomal enlargement serves as one of the earliest manifestations of AD and AD-DS. The link to decreased retrograde axonal transport and signaling of NGF, and atrophy of BFCNs (Xu et al., 2016) points to regulation of Rab5 activity as a rational treatment target. A Rab5-based strategy would benefit AD and AD-DS by restoring axonal transport of NGF-containing signaling endosomes as well as transport of other axonal cargoes impacted by excessive activation of Rab5. A number of approaches can be considered, including the discovery of small molecules that impact GTP loading of Rab5 as well as RNA-based approaches, including CNS delivery of specific ASOs.

## Summary

The foregoing makes clear the complexity of AD. In spite of this, we are of the optimistic view that existing insights may be sufficient for launching a first wave of disease-modifying treatments. Nevertheless, advances in genetics and cellular biology will be needed to further enhance our understanding of pathogenesis and the mechanisms that must be targeted for the most robustly effective treatments. Especially important will be efforts to define the structure, function and location of toxic species of Aβ and tau, including biomarkers to announce their presence and interventions to combat their production, accumulation, or actions. A focus on detecting the earliest manifestations in selectively vulnerable populations, including BFCNs, would augment progress. In particular, we are intrigued by the intersection of Aβ- and tau-mediated pathology in dysregulation of the endosomal pathway and disordered transport of neurotrophic factor signaling as playing a defining role in neuron dysfunction and death. Finally, we anticipate continued benefit from asking how the various hypotheses for AD interact and reinforce one another.

## Author Contributions

X-QC and WM wrote and edited the manuscript and approved it for publication.

## Conflict of Interest Statement

The authors declare that the research was conducted in the absence of any commercial or financial relationships that could be construed as a potential conflict of interest.
